# Low prevalence of *CCR5*-*Δ32, CCR2*-*64I* and *SDF1*-*3′A* alleles in the Baiga and Gond tribes of Central India

**DOI:** 10.1186/s40064-015-1238-6

**Published:** 2015-08-25

**Authors:** Deepak Bharti, Ashish Kumar, Ranjeet Singh Mahla, Sushil Kumar, Harshad Ingle, Tushar Yadav, Anamika Mishra, Ashwin Ashok Raut, Himanshu Kumar

**Affiliations:** Laboratory of Immunology, Department of Biological Sciences, Indian Institute of Science Education and Research (IISER), Indore-Bypass Road, Bhauri, Bhopal, 460066 India; Chemical Engineering Department, Sardar Vallabhbhai National Institute of Technology, Surat, 395007 India; Pathogenomics Lab, ICAR-National Institute of High Security Animal Diseases, Anand Nagar, Bhopal, 462022 India; Laboratory of Host Defense, WPI Immunology Frontier Research Center, Osaka University, Osaka, Japan

**Keywords:** Primitive tribes, Non-primitive tribes, Chemokine receptors, HIV-1 resistant polymorphisms, Relative hazard

## Abstract

Human immunodeficiency virus-1 (HIV-1) which causes acquired immune deficiency syndrome (AIDS), by infecting CD4^+^ immune cells and hence weakening the host defense mechanism till death, is one of the major factor responsible for human demises worldwide. Both innate (monocytes and macrophages) and adaptive (T cells) immune cells expresses chemokines receptors (2 and 5) and stromal cell derived factor-1 (*SDF*-*1*) which play crucial role in HIV-1 virus entry and progression. Allele variants of genes *CCR5* (*CCR5*-*Δ32*), *CCR2* (*CCR2*-*64I*) and *SDF1* (*SDFA*-*3*′*A*; the ligand of *CXCR4*) are known to slow down the HIV-1 progression in infected individual. In the present study, the frequency of *CCR5*-*Δ32, CCR2*-*64I* and *SDF1*-*3*′*A* alleles in primitive tribe (Baiga) and a non-primitive tribe (Gond) of central India were investigated. A total 200 seronegative samples for HIV from healthy individuals of tribes were analyzed and observed allele frequencies of *CCR5*-*Δ32, CCR2*-*64I* and *SDF1*-*3*′*A* were (0, 0.035, 0.080) and (0, 0.110, 0.100) in Baiga and Gond respectively. Minor allele frequency of these alleles of Gond and Baiga tribes were compared with different populations of the world for relative hazard (RH), which indicate the risk of progression after infection of HIV1. The RH values were calculated based on genotypic frequency, showed the high RH value (RH1-AIDS1993-0.98, RH2-AIDS1987-0.98 and death/RH3-0.97) in Baiga tribe, indicates the low level of resistance against HIV-1 progression after infection.

**Electronic supplementary material:**

The online version of this article (doi:10.1186/s40064-015-1238-6) contains supplementary material, which is available to authorized users.

## Background

It has been nearly three to four decade since the report of the first acquired immune deficiency syndrome (AIDS) case which attracted the world’s attention. The AIDS is caused by the human immunodeficiency virus (HIV) which targets various types of cells of host immunity such as dendritic cells, macrophages and T cell subtypes etc. and slowly weakens the host immune system leading to severe immunodeficiency. According to recent report, about 70 million people have been infected by the HIV which caused 35 million deaths worldwide (Horvath et al. 2012; Ruelas and Greene 2013). HIV-1 and HIV-2 are the two types of HIV (McCutchan 2006), of which, the former is found in majority of the AIDS cases and is considered to be highly pathogenic. Upon infection, progress of HIV-1 has been shown to be influenced by C–C family chemokine receptors (CCR) like CCR5, CCR2 and SDF1 (a ligand of CXCR4). These molecules have been shown to play an important role in the entry of HIV-1 into various cell types such as macrophages, monocytes and T-cells (CD4^+^) (Herbein and Varin 2010; Doitsh et al. 2014). A 32 bp deletion in CCR5 coding sequence is well known as *CCR5*-*Δ32* polymorphism, was identified in case of near complete resistance from HIV1 in homozygous state and slower progression of HIV1 in heterozygote state (Dean et al. 1996; Liu et al. 1996; Samson et al. 1996). Many populations studies conducted worldwide have been shown that genetic variants *CCR5*-*Δ32* (32-bp deletion), *CCR2*-*64I* (V → I) and *SDF1*-*3*′*A* (G-801A) slower the rate of HIV-1 progression thus leading to delayed onset and reduced severity of AIDS. The risk of AIDS onset for populations is calculated through determination of relative hazard (RH) based on the occurrence of mutation in these three gene loci (Dean et al. 1996; Smith et al. 1997; Winkler et al. 1998).

India has the largest portion of the world’s primitive and non-primitive tribal populations, among which most of the tribal populations distributed mainly in eastern and central Indian states followed by Rajasthan and Gujarat states of India. According to census of 2011, the total populations of India estimated are 1.29 billion in which tribal populations contributed 0.1 billion. Tribal populations are distributed all over India except Punjab and Haryana, states of India. A significant part (14.7 %) of Indian tribal populations is distributed in the central Indian state Madhya Pradesh. A tribe is a group of people who are linguistically, socially and geographically isolated from modern human populations and for their livelihood, they are dependent on their land while primitive tribes are generally considered as those people who are isolated from tribes in past and are more backward with very low income, lived in difficult areas in small and scattered habitat therefore their social interaction with main stream is almost negligible. Baiga tribe is more geographically and socially isolated than Gond tribe from other caste populations of India. Consequently, there is less chance of Baiga tribe to share their gene pool with well developed human caste populations. Due to the same reason, on the basis of primitive and non primitive, we have selected Baiga and Gond tribes for present study. Baiga is a primitive tribe (Population size: approximately 0.5 million) mostly found in Mandla and Balaghat districts of a central Indian state, Madhya Pradesh. They practice consanguineous marriage and remain poorly informed about various infectious diseases including HIV/AIDS (Reddy and Modell 1997; Saha et al. 2013). Women of the tribe are known to sporting tattoos on their body using needles. The Gond is the tribal community mostly found in the forests of the central India. According to census 2011 Gond is a second largest tribe in Madhya Pradesh, a central Indian state with a population of 4.36 million. They are widely spread in the Chhindwara district of Madhya Pradesh, Bastar district of neighbouring Indian states of Chhattisgarh and also in parts of Maharashtra, Andhra Pradesh and Orissa states. The name by which the Gond calls themselves is Koi or Koitur which means unclear. They are one of the largest tribal groups in the world.

To date, no genetic studies have been conducted on chemokine marker polymorphism which related with HIV infection risk in primitive Baiga tribe and non-primitive Gond tribe.

## Results

*CCR5*-*Δ32* mutant is well known to provide resistance from HIV-1 by preventing cell entry through expression of truncated protein. Therefore, individuals harbouring homozygous mutant allele (Δ32/Δ32) are highly resistant to HIV-1 infection whereas, heterozygous (Δ32/wt) have partial protection (Su et al. 2000). In this study, deletion mutant genotype (Δ32/Δ32) as well as (Δ32/wt) were not observed in both the tribal populations; (Table 1). *CCR2*-*64I* and *SDF1*-*3*′*A* mutant alleles are also shown to be associated with suppression of HIV-1 progression to AIDS; however, the suppressive effect is lower in comparison to *CCR5*-*Δ32*. The suppressive effect exerted by the *SDF1*-*3*′*A* mutation is recessive i.e., observed only in homozygote mutant (3′A/3′A) (Su et al. 1999). The frequency of *SDF1* genotype (3′A/3′A) was found to be very less (1 %) in Gond and was not found in Baiga tribe. The *CCR2* genotype (64I/64I) was not found in Baiga tribe; however, it is present in Gond tribe with a very low frequency (1 %) (Table 1). Additionally, the frequency of heterozygous (64V/64I) was observed less in Baiga (7 %) compared to the Gond tribe (20 %) (Table 1). Further analysis of genotype data did not show significant deviation from the Hardy–Weinberg expected frequency, indicating that the alleles are in genetic equilibrium (Table 1). Furthermore, we estimated the RH indices by using the three locus genotype data. RH values were calculated for all the three definitions, AIDS-1993, AIDS-1987 and Death by using formula RH = ∑(Wi*Pi); where *Wi* and *Pi* denotes the genotype specific RH and frequencies respectively. RH value vary from population to population, however geographically or ethically related populations tend to have similar RH values as they have comparable minor allele frequency (MAF) for three genes. Out of 27 possible three locus genotypes, we found only 4 in Baiga and 6 in Gond tribe (Additional file 1: Table S1). Detection of only 4 and 6 different genotypes is due to the fact that the *CCR5*-*wt* allele is fixed in these populations and also the homozygous genotypes of *CCR2* genotype (*64I/64I*) and *SDF1* (*3*′*A/3*′*A*) are not found in the Baiga population. Baiga tribe showed a high RH value [AIDS1993-0.98 (RH1), AIDS1987-0.98 (RH2) and Death-0.97 (RH3)] (Table 2).

**Table 1:** Distribution of genotype and allele frequency of *CCR5*, *CCR2* and *SDF*-*1* genes in primitive tribe (Baiga) and non-primitive tribe (Gond) of Central India

Sr. No.	Tribe	N	*CCR5*				*CCR2*					*SDF1*				
			Genotype		MAF	χ^2^ (H.W.)	Genotype			MAF	χ^2^ (H.W.)	Genotype			MAF	χ^2^ (H.W.)
			CCR5	Δ32			GG	AG	AA			GG	AG	AA		
1.	Baiga	100	100	0	0	–	93	7	0	0.035	0.720	84	16	0	0.080	0.390
2.	Gond	100	100	0	0	–	79	20	1	0.110	0.830	82	17	1	0.100	0.910

Major alleles for *CCR5*, *CCR2* and *SDF1* are wild type (wt), “G” and “G” respectively. Minor alleles for *CCR5, CCR2* and *SDF1* are “Δ32” (*CCR5*-*Δ32*), “A” (V → I) and “A” (*SDF1*-*3*′*A*) respectively

*MAF* and *H.W.* represents minor allele frequency and Hardy–Weinberg respectively

**Table 2:** The RH values in Baiga and Gond tribes of Central India

Population	N	RH1	RH2	RH3
Baiga	100	0.98	0.98	0.97
Gond	100	0.92	0.92	0.90

The RH values were calculated based on three AIDS definitions, AIDS-1993 (RH1), AIDS-1987 (RH2), and Death (RH3)

## Discussion

**Fig. 1:**
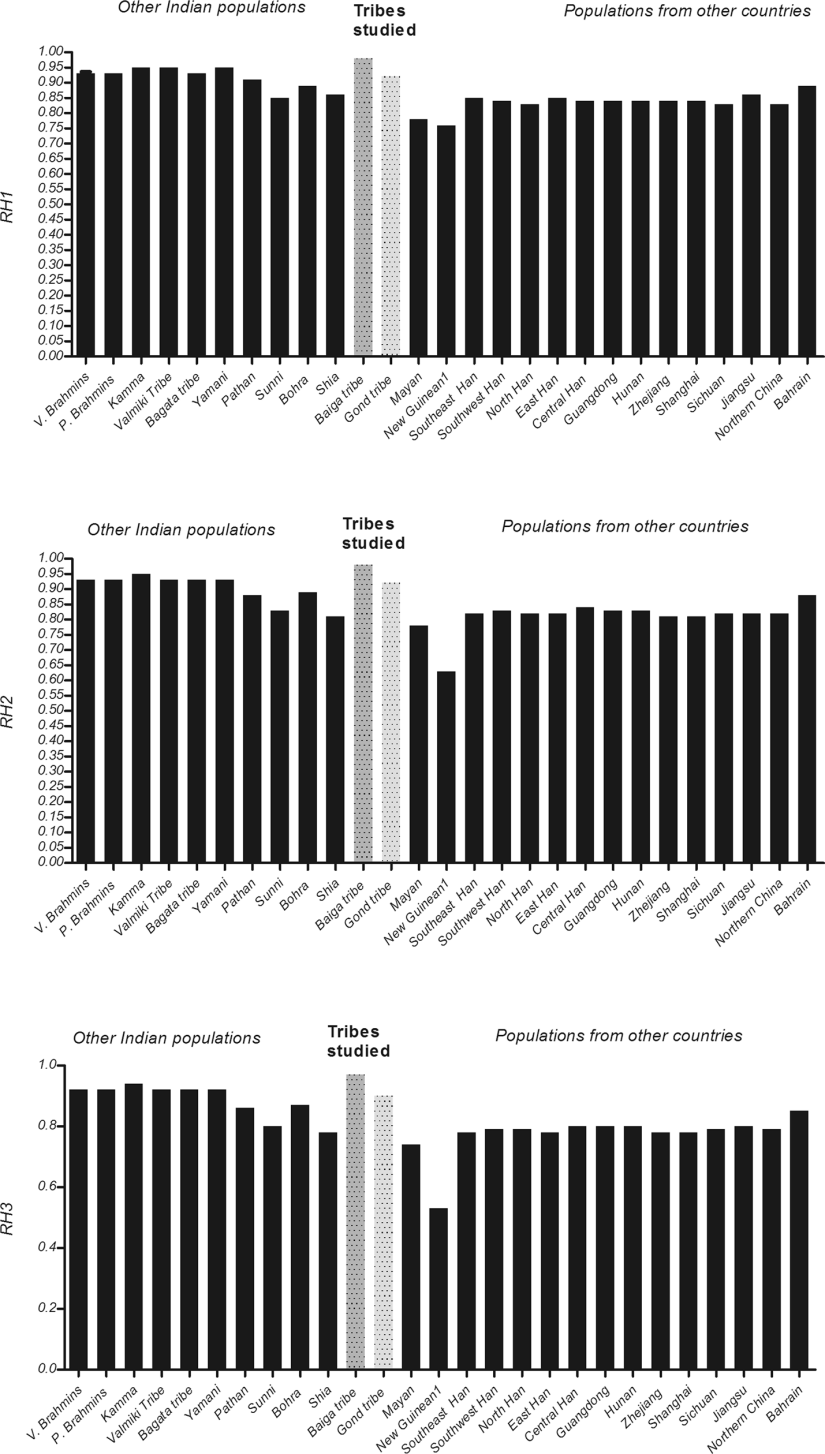
Comparison of relative hazard (RH) in Baiga tribe with Gond tribe and rest of world populations (Ramana et al. 2001; Salem et al. 2009; Su et al. 2000; Xiao et al. 2000). RH1, RH2 and RH3 refer to AIDS-1993, AIDS-1987 and Death respectively

HIV-1 is highly pathogenic and relatively modern virus compared to the several other pathogens. Progression of HIV-1 after infection in slow in those individuals who carries the mutant form of genes such as *CCR5, CCR2* and *SDF1*. These mutational changes have originated outside India, however, due to social interaction among various world populations, the frequency of mutant alleles were raised in several populations. Earlier study on ethnic populations of India have shown that *CCR5*-*Δ32* allele is completely absent in tribes, however it can be found very low in Caste populations (Majumder and Dey 2001). It can be predicted that endogamy practices, geographical isolation might be the factors for the low frequencies of *CCR5*-*Δ32, CCR2*-*64I* and *SDF1*-*3*′*A.* Due to the absence of social interactions with modern populations, primitive tribes have not acquired the alleles that reduce the progression of HIV-1 infection making them highly susceptible. In this study all the individuals were expressing homozygous wild type allele (wt/wt) for the *CCR5* gene (Table 1), indicating that the allele *CCR5*-*Δ32* is completely absent in both the tribal groups and very low allele frequency of mutant alleles of *CCR2* and *SDF1* were recorded in Gond and Baiga tribes. All together our study indicates that the frequency of the alleles, *CCR5*-*Δ32, CCR2*-*64I* and *SDF1*-*3*′*A* are significantly low in both the tribal populations (Table 1). The comparison of RH values of present studied tribe with earlier studied populations of India and the different populations (Su et al. 2000; Xiao et al. 2000; Ramana et al. 2001 and Salem et al. 2009) of the world showing the highest RH value in primitive tribe “Baiga” (Fig. 1; Additional file 1: Table S2).

## Conclusion

The frequency of *CCR5*-*Δ32, CCR2*-*64I* and *SDF1*-*3*′*A* are recorded low in Gond and very low in the Baiga tribe. It can be predicted that endogamy practices, geographical isolation might be the factors for low frequencies of *CCR5*-*Δ32, CCR2*-*64I* and *SDF1*-*3*′*A*. Due to absence of social interaction with modern populations, primitive tribes have not acquired the alleles that reduce the progression of HIV-1 infection making them highly susceptible to the same. The high RH of AIDS onset indicates very low resistance in Baiga against HIV-1 progression after infection. Therefore, present study showed that there are not enough protective shields against HIV-1 for central Indian tribes.

## Methods

The 200 samples were obtained from unrelated healthy individuals of Baiga and Gond tribes of central India. All the samples were seronegative for HIV. This study was carried out according to the ethical guidelines of Institutional Ethical Committee (IEC), IISER Bhopal and with the written consent of all the participants. Blood samples from the individuals were spotted on Whatman FTA classic Cards (GE healthcare) and processed for PCR as per manufacturer instructions. The region containing the *CCR5*-*Δ32, CCR2*-*64I* and *SDF1*-*3*′*A* were amplified using Phusion Blood Direct PCR Kit (Thermo scientific) as per manufacturer protocol with corresponding Primers (*CCR5*-Fw: 5′-GCTGTCGTCCATGCTGTGTTT-3′, Rv:5′-CAACCTGTTAGAGCTACTGCAATT-3′); (*CCR2*-Fw:5′ATCAGAAATACCAACGAGAGCGG-3′, Rv:5′-ACACCGAAGCAGGGTTTTCAGG-3′) and (*SDF1*-Fw:5′-CAGTCAACCTGGGCAAAGCC-3′, Rv:5′-AGCTTTGGTCCTGAGAGTCC-3′) (Struyfa et al. 2000; Junhua et al. 2000; Bhatnagar et al. 2009). The sequencing of PCR products were performed using 3730 DNA Analyzer (Applied Bio systems) sequencer using the Fw primer used in the PCR amplification. The SNPs were then analysed using Sequencing Analysis software v5.4. Allele frequencies were calculated using the formula, Allele Frequency = {(2 × Number of individuals having genotype homozygous for that particular allele) + (Number of individuals having heterozygous genotype)}/(2 × Total Number of individuals). RH values is estimated on based on genotype frequency and calculated for all the three definitions, AIDS-1993, AIDS-1987 and Death by using formula RH = ∑(Wi*Pi); where *Wi* and *Pi* denote the genotype specific RH and frequencies respectively.  

## Additional file

Additional file 1:
**Table S1.** The relative hazard (RH) value calculations based on the three-locus genotype for each individual of Primitive tribe, Baiga and Non primitive tribe, Gond. **Table S2.** The relative hazard values of the other populations of the world.
