# Supplementary material for: Low prevalence of CCR5-Δ32, CCR2-64I and SDF1-3′A alleles in the Baiga and Gond tribes of Central India
Source: Springerplus. 2015 Aug 25;4:451. doi: 10.1186/s40064-015-1238-6 (PMC4547972; doi:10.1186/s40064-015-1238-6)
Supplement: Additional file 1: — Table S1. The relative hazard (RH) value calculations based on the three-locus genotype for each individual of Primitive tribe, Baiga and Non primitive tribe, Gond. Table S2. The relative hazard values of the other populations of the world. [file 40064_2015_1238_MOESM1_ESM.doc]

**Online supplementary Information**

**Table S1:**  Relative hazard (RH) value; based on the three-locus genotype of each individual of Primitive tribe, Baiga and Non primitive tribe, Gond.

| **Baiga Tribe** | | | | | | | | | | | | | | |
| --- | --- | --- | --- | --- | --- | --- | --- | --- | --- | --- | --- | --- | --- | --- |
| **Sr. No.** | **Observed Three-locus Genotypes**  **(*CCR5*-*CCR2*-*SDF1*)** | **Observed**  **Genotype Frequency based on three locus Genotype**  **(*Pi*)** | | **The genotype-specific RH1, AIDS-1993**  **(*Wi*)** | **The genotype-specific RH2 ,**  **AIDS-1987**  **(*Wi*)** | | **The genotype-specific RH3 ,Death**  **(*Wi*)** | | **RH1**  **(*WiPi*)** | | **RH2**  **(*WiPi*)** | | | **Death**  **(*WiPi*)** |
| 1 | AA-BB-CC | 0.84 | | 1 | 1 | | 1 | | 0.8400 | | 0.8400 | | | 0.8400 |
| 2 | AA-BB-Cc | 0.09 | | 1 | 1 | | 1 | | 0.0900 | | 0.0900 | | | 0.0900 |
| 3 | AA-Bb-Cc | 0.03 | | 0.65 | 0.66 | | 0.60 | | 0.0195 | | 0.0200 | | | 0.0180 |
| 4 | AA-Bb-CC | 0.04 | | 0.65 | 0.66 | | 0.60 | | 0.0260 | | 0.0270 | | | 0.0240 |
| Total relative hazard (RH = *∑WiPi*) | | | | | | | | | **0.98** | | **0.98** | | | **0.97** |
| **Gond Tribe** | | | | | | | | | | | | | | |
| **Sr. No.** | **Observed Three-locus Genotypes**  **(*CCR5-CCR2-***  ***SDF1*)** | | **Observed**  **Genotype Frequency**  **based on three locus Genotype**  **(*Pi*)** | **The genotype-specific RH1, AIDS-1993**  **(*Wi*)** | | **The genotype-specific RH2 ,**  **AIDS-1987**  **(*Wi*)** | | **The genotype-specific RH3 ,Death**  **(*Wi*)** | | **RH2**  **(*WiPi*)** | | **RH1**  **(*WiPi*)** | **RH2**  **(*WiPi*)** | |
| 1 | AA-BB-CC | | 0.63 | 1 | | 1 | | 1 | | 0.6300 | | 0.6300 | 0.6300 | |
| 2 | AA-BB-Cc | | 0.14 | 1 | | 1 | | 1 | | 0.1400 | | 0.1400 | 0.1400 | |
| 3 | AA-Bb-Cc | | 0.03 | 0.65 | | 0.66 | | 0.60 | | 0.0195 | | 0.0198 | 0.0180 | |
| 4 | AA-Bb-CC | | 0.18 | 0.65 | | 0.66 | | 0.60 | | 0.1170 | | 0.1188 | 0.1080 | |
| 5 | AA-bb-CC | | 0.01 | 0.65 | | 0.66 | | 0.60 | | 0.0065 | | 0.0066 | 0.0060 | |
| 6 | AA-Bb-cc | | 0.01 | 0.63 | | 0.35 | | 0.23 | | 0.0063 | | 0.0035 | 0.0023 | |
| Total relative hazard (RH = ∑*WiPi*) | | | | | | | | | | **0.92** | | **0.92** | **0.90** | |

A, B and C refer to the wild type alleles of *CCR5, CCR2* and *SDF1*, respectively; and a, b and c are mutant alleles at the respective loci.

**Table S2:** RH values for Baiga, Gond and worldwide populations having population size of at least 40.

| **Country** | **Population** | **N** | **RH1** | **RH2** | **RH3** | **Reference** |
| --- | --- | --- | --- | --- | --- | --- |
| India | V. Brahmins | 59 | 0.94 | 0.93 | 0.92 | Ramana, G.V. et al.2001 |
| India | P. Brahmins | 45 | 0.93 | 0.93 | 0.92 | Ramana, G.V. et al.2001 |
| India | Kamma | 51 | 0.95 | 0.95 | 0.94 | Ramana, G.V. et al.2001 |
| India | Valmiki Tribe | 42 | 0.95 | 0.93 | 0.92 | Ramana, G.V. et al.2001 |
| India | Bagata tribe | 45 | 0.93 | 0.93 | 0.92 | Ramana, G.V. et al.2001 |
| India | Yamani | 49 | 0.95 | 0.93 | 0.92 | Ramana, G.V. et al.2001 |
| India | Pathan | 50 | 0.91 | 0.88 | 0.86 | Ramana, G.V. et al.2001 |
| India | Sunni | 50 | 0.85 | 0.83 | 0.8 | Ramana, G.V. et al.2001 |
| India | Bohra | 49 | 0.89 | 0.89 | 0.87 | Ramana, G.V. et al.2001 |
| India | Shia | 50 | 0.86 | 0.81 | 0.78 | Ramana, G.V. et al.2001 |
| **India** | **Baiga tribe** | **100** | **0.98** | **0.98** | **0.97** | **Present Study** |
| **India** | **Gond tribe** | **100** | **0.92** | **0.92** | **0.90** | **Present Study** |
| America | Mayan | 40 | 0.78 | 0.78 | 0.74 | Su, B. et al.2000 |
| Oceania | New Guinean1 | 69 | 0.76 | 0.63 | 0.53 | Su, B. et al.2000 |
| China | Southeast Han | 40 | 0.85 | 0.82 | 0.78 | Su, B. et al.2000 |
| China | Southwest Han | 48 | 0.84 | 0.83 | 0.79 | Su, B. et al.2000 |
| China | North Han | 138 | 0.83 | 0.82 | 0.79 | Su, B. et al.2000 |
| China | East Han | 407 | 0.85 | 0.82 | 0.78 | Su, B. et al.2000 |
| China | Central Han | 168 | 0.84 | 0.84 | 0.8 | Su, B. et al.2000 |
| China | Guangdong, Guangxi, Fujian, Hainan | 89 | 0.84 | 0.83 | 0.8 | Xiao, J. et al. 2000 |
| China | Hunan, Hubei, Jiangxi, Anhui | 191 | 0.84 | 0.83 | 0.8 | Xiao, J. et al. 2000 |
| China | Zhejiang | 183 | 0.84 | 0.81 | 0.78 | Xiao, J. et al. 2000 |
| China | Shanghai | 116 | 0.84 | 0.81 | 0.78 | [Xiao, J. et al.](#_ENREF_3) 2000 |
| China | Sichuan | 48 | 0.83 | 0.82 | 0.79 | Xiao, J. et al. 2000 |
| China | Jiangsu | 190 | 0.86 | 0.82 | 0.8 | [Xiao, J. et al.](#_ENREF_3) 2000 |
| China | Northern China | 174 | 0.83 | 0.82 | 0.79 | [Xiao, J. et al.](#_ENREF_3) 2000 |
| Bahrain | Bahrain | 304 | 0.89 | 0.88 | 0.85 | Salem, A.H.et al.  2009 |

N indicates number of samples.

RH1, RH2 and RH3 refer to AIDS-1993, AIDS-1987 and Death respectively.
